# The value of cerebrospinal fluid cytology in the diagnosis of atypical medulloblastoma: a case report and review of the literature

**DOI:** 10.3389/fmed.2025.1641160

**Published:** 2025-10-20

**Authors:** Shaoqiang Xu, Lina Cheng, Chunxia Huang, Yuanyang Ye, Keyuan Lai

**Affiliations:** ^1^Department of Clinical Laboratory, Guangdong Sanjiu Brain Hospital, Guangzhou, China; ^2^Department of Radiology, Guangdong Sanjiu Brain Hospital, Guangzhou, China

**Keywords:** medulloblastoma, cerebrospinal fluid cytology, atypical imaging, leptomeningeal dissemination, diagnostic value

## Abstract

Medulloblastoma is a highly aggressive malignant tumor of the central nervous system in children, and early diagnosis is crucial for improving prognosis. In this article, we report a case of an 8-year-old male patient who presented with intermittent headache and vomiting, and whose cranial MRI showed subcerebellar tonsillar herniation with hydrocephalus, but lacked the typical features of tumor enhancement and was misdiagnosed as meningitis. After obtaining a cerebrospinal fluid specimen via lumbar puncture, tumor cells were found in it, which led to the diagnosis of medulloblastoma. This study provides a practical model for the differential diagnosis of atypical medulloblastoma on imaging and highlights the irreplaceable role of cerebrospinal fluid cytology in the identification of tumor metastasis.

## 1 Introduction

Medulloblastoma, a highly malignant embryonic neuroepithelial tumor originating from the cerebellum or fourth ventricle, is one of the most prevalent childhood brain tumors, accounting for approximately 20% of cases (1, 2). Its diagnosis is challenging due to its aggressive nature and propensity for leptomeningeal dissemination (LMD) throughout the craniospinal axis (3). Magnetic resonance imaging (MRI), with its multiparametric capabilities and comprehensive anatomical coverage, serves as the primary imaging modality for suspected cases (4). However, in instances with atypical MRI findings, supplementary diagnostic methods are essential (5, 6). Cerebrospinal fluid (CSF) cytology is widely regarded as the gold standard for detecting subclinical LMD, offering critical diagnostic value that complements imaging (7). Consequently, CSF cytology plays a crucial role not only in the initial diagnosis but also in disease monitoring and prognostic assessment for medulloblastoma, a tumor known for its cerebrospinal fluid-borne metastases (8–10). It effectively compensates for the limitations of MRI, particularly in patients with atypical imaging presentations (11, 12). This study details a pediatric case of medulloblastoma with an atypical initial MRI, in which CSF cytology was pivotal in establishing the definitive diagnosis. Furthermore, we present a review of the relevant literature and propose a refined diagnostic framework to underscore the integrative role of cytological and molecular analysis in such challenging scenarios.

## 2 Case reports

**Figure 1:**
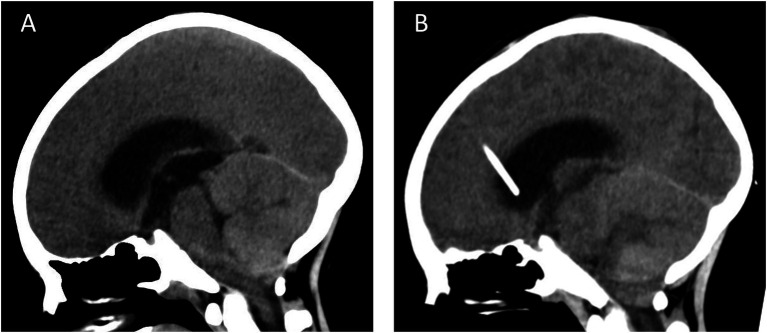
CT examination **(A)** the cerebellar tonsils moved downward beyond the greater occipital foramen to the atlantoaxial level, and the diagnosis of subcerebellar tonsillar hernia combined with supratentorial obstructive hydrocephalus was made; **(B)** on examination after the emergency Ommaya capsule placement and drainage surgery, the subcerebellar tonsillar hernia and supratentorial hydrocephalus had not yet been relieved, and the placement of a drainage tube was seen in the lateral ventricle.

The patient, a male, 8 years old, entered another hospital with intermittent headache with nausea and vomiting, and a cranial MRI showed cerebellar swelling in the posterior cranial fossa, which was initially diagnosed as meningitis. However, after symptomatic treatment, the symptoms did not improve significantly, so he was referred to our hospital for further evaluation and treatment. Upon admission to our hospital, physical examination showed that the patient had no fever, poor mental status, and no clear neurologic abnormality. Computed tomography angiography (CTA) findings suggested a subcerebellar tonsillar hernia (Figure 1A) with severe hydrocephalus. Based on the imaging findings and combined with the MRI images provided by the patient’s family, it was decided to perform emergency Ommaya bursa placement and drainage on the same day after comprehensive evaluation to relieve intracranial pressure. Postoperative follow-up cranial CT showed no significant improvement in hydrocephalus, and no other abnormalities were detected (Figure 1B).

**Figure 2:**
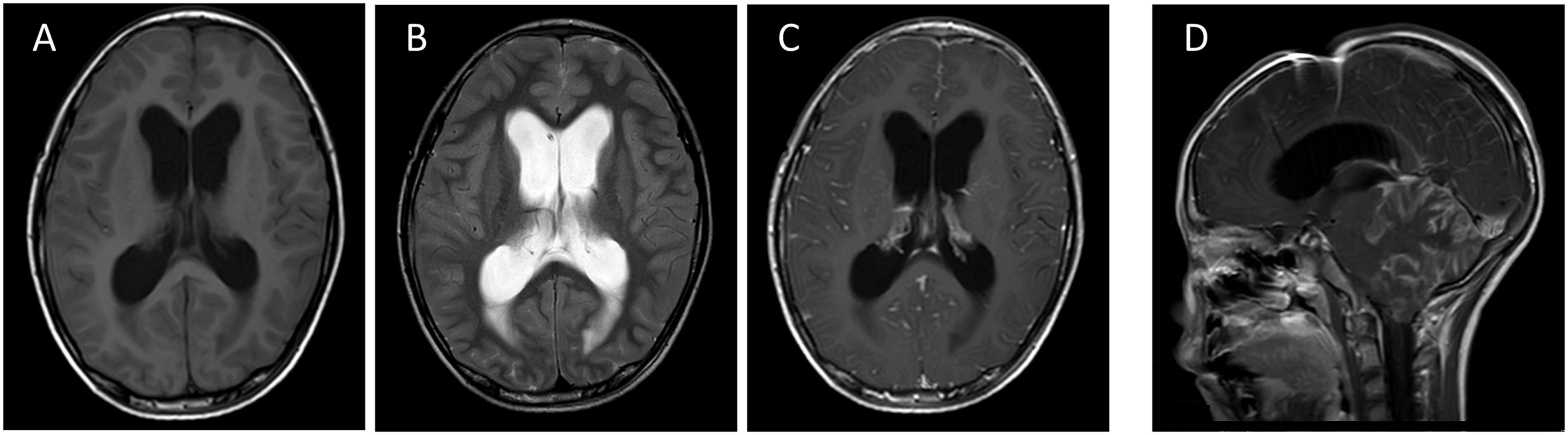
Horizontal T1 **(A)**, horizontal T2 **(B)** and horizontal T1 enhancement **(C)** images demonstrate enlargement of the supratentorial ventricular system suggestive of hydrocephalus, with extensive gyrus-like thickening and enhancement of the soft meninges predominantly present in the cerebellar hemispheres, cerebellar vermis, and brainstem bilaterally, which is combined with multiple abnormal linear enhancements of the supratentorial soft meninges, suggesting a high likelihood of meningitis. Sagittal T1 **(D)** still shows subcerebellar tonsillar herniation, supratentorial obstructive hydrocephalus and its post-drainage manifestations.

MRI revealed multiple predominantly meningeal-based enhancing lesions with diffuse abnormal signals in the bilateral cerebellar hemispheres, cerebellar vermis, and brainstem, initially suggesting a high likelihood of meningitis. Despite the extensive involvement, no definite focal space-occupying lesion was identified in the cerebellar vermis, and this atypical presentation posed the primary diagnostic challenge (Figures 2A–D).

**Figure 3:**
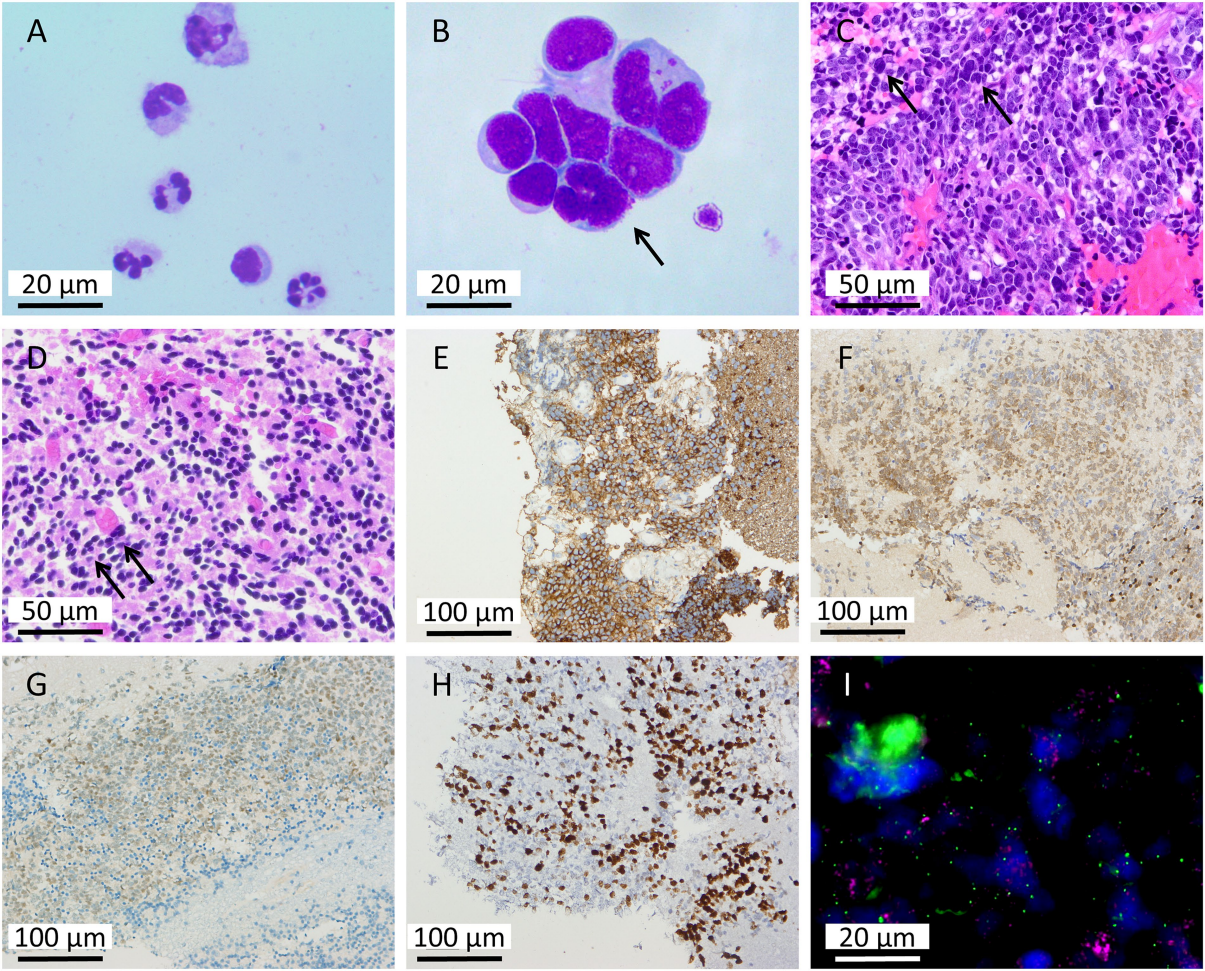
Cytological, histopathological, and molecular pathological findings of the medulloblastoma. **(A,B)** CSF cytology. **(A)** The initial CSF specimen (from hydrocephalus) showed a predominance of neutrophils with no atypical cells identified (Giemsa staining; ×1,000). **(B)** The second CSF specimen (from lumbar puncture) revealed atypical cells resembling medulloblastoma cells (black arrow, Giemsa staining; ×1,000). **(C)** Intraoperative frozen section examination revealed sheets of small, round, poorly differentiated tumor cells. These cells were densely packed with enlarged, oval to irregular nuclei. Occasional mitotic figures were noted, and some cells exhibited small nucleoli. Apoptosis was readily observed, while no definitive necrosis was identified. (HE staining; ×400). **(D)** Postoperative pathological examination revealed multifocal aggregates of small, round, poorly differentiated tumor cells. The neoplastic cells were densely packed and exhibited enlarged, oval to irregular nuclei with a high nuclear-to-cytoplasmic (N/C) ratio. Mitotic figures were readily identified, and some cells contained small nucleoli. No definitive necrosis was observed. (HE staining; ×400). **(E–G)** Immunohistochemical analysis. **(E)** Synaptophysin (Syn) positivity (×400). **(F)** Weak NeuN immunoreactivity (×400). **(G)** SOX-10 expression (×400). **(H)** The tumor cells exhibited a high Ki-67 proliferation index of up to 40% in hotspot regions (×400). **(I)** Fluorescence *in situ* hybridization (FISH) showed positive MYC gene amplification (×1,000).

Doctors utilized shunted hydrocephalus samples to send for testing to clarify the etiology. Despite a high clinical suspicion of infectious or autoimmune encephalitis, further testing failed to provide definitive diagnostic clues. CSF cytologic analysis detected only a small number of neutrophils (Figure 3A), and biochemical tests showed decreased levels of protein, lactate dehydrogenase (LDH), and lactate (Table 1). Pathogenetic testing did not detect pathogenic pathogens, and the autoimmune encephalitis antibody profile test results are normal. Common diseases such as tuberculosis infection, cryptococcal infection, viral infection and autoimmune meningoencephalitis were thus excluded. As the initial examination failed to identify the cause of the disease, and the patient’s symptoms remained unrelieved with recurrent headaches and vomiting, the Laboratory Department considered it necessary to rule out the possibility of a neoplastic lesion based on the clinical manifestations. Since the collection of CSF from the shunt would affect the results of the examination, the laboratory recommended a lumbar puncture to review the CSF cytology to further assist in the diagnosis. A 2–3 mL aliquot of cerebrospinal fluid was placed into a specialized CSF collection tube and centrifuged in a cytocentrifuge at 68 × g for 10 min. Following centrifugation, the supernatant was absorbed by filter paper, leaving the cellular components concentrated on a glass slide. After air-drying, the smear was stained with Giemsa stain for 15 min. Finally, the slides were independently examined and reported by two experienced laboratory technologists. Atypical cells are characterized by a spectrum of morphological abnormalities, including: cellular pleomorphism and anisocytosis; nuclear features such as karyomegaly, anisokaryosis, marked nuclear pleomorphism, and conspicuous single or multiple nucleoli. Furthermore, atypical cells of different origins exhibit distinct morphological characteristics. This examination revealed the presence of atypical cells in the CSF (Figure 3B), and based on the cellular morphology, this was considered as the possibility of medulloblastoma cells. The results of nucleated cell counts and biochemical tests were significantly abnormal compared with the previous ones (Table 1), which provided key clues to the clinical diagnosis and further clarified the direction of the disease. During further examination and diagnosis, the patient underwent posterior fossa mass resection combined with decompressive craniectomy. The intraoperative frozen section pathology indicated a small round cell malignant tumor (Figure 3C). The subsequent definitive histopathological examination confirmed the diagnosis of medulloblastoma with an anaplastic large cell variant (Figure 3D). Immunohistochemical analysis supported the tumor’s neuroectodermal origin, demonstrating synaptophysin positivity, weak NeuN immunoreactivity, and SOX-10 expression (Figures 3E–G). The tumor cells exhibited high proliferative activity with a Ki-67 proliferation index reaching 40% in hotspot regions (Figure 3H). Fluorescence *in situ* hybridization revealed MYC gene amplification (Figure 3I) while MYCN amplification was absent. The combination of MYC amplification as a well-established poor prognostic indicator and the presence of anaplastic histomorphology strongly suggested classification within Group 3 medulloblastoma. Despite postoperative clinical stabilization, the family elected to forgo adjuvant chemotherapy and arranged for hospital discharge after a comprehensive understanding of the high-risk diagnosis.

**Table 1:** Routine cerebrospinal fluid and biochemical tests.

Test	First examination	Second examination	Reference range
Total cell count (× 10^6^/L)	85	25	0 ~ 5
Erythrocyte count (× 10^6^/L)	2	8	Absent
Nucleated cell count (× 10^6^/L)	83	17	0 ~ 5
Nucleated cell classification			
%Lymphocytes	10	70	—
%Monocytes	12	14	—
%Neutrophils	78	4	—
%Heterogeneous cells	—	12	—
Protein (g/L)	0.1	4.4	0.15 ~ 0.45
Aspartate aminotransferase (U/L)	1.4	42.1	0 ~ 19.0
Adenosine deaminase (U/L)	0.5	1.2	0 ~ 8.0
Chloride (mmol/L)	131.0	121.7	120.0 ~ 130.0
Glucose (mmol/L)	4.0	2.3	2.5 ~ 4.5
Lactate Dehydrogenase (U/L)	7.5	220.1	10.0 ~ 25.0
Lactate (mmol/L)	1.3	3.53	1.3 ~ 1.9

## 3 Discussion

Medulloblastoma, one of the most common malignant brain tumors in children, unfortunately presents with metastases at diagnosis in over 40% of patients, carrying a grim median survival (13, 14). As a non-invasive imaging method, MRI is able to provide clear images of brain structures and effectively assess the location, size, morphology, and relationship with surrounding structures of the tumor, providing key clues for the initial diagnosis (15). However, MRI has limitations in sensitivity, as it can miss early microscopic lesions, leading to atypical manifestations in about 20–30% of patients (7, 16). Consequently, for patients with suspected meningeal or spinal cord involvement, CSF cytology serves as a necessary adjunctive diagnostic tool (17, 18).

In this case, the patient’s symptoms manifested as intermittent headache, nausea, and vomiting, and initial imaging showed abnormal signals in the cerebellum and brainstem, suggesting possible meningitis, but further imaging failed to confirm this diagnosis. After a series of empiric treatments, the patient’s symptoms did not improve significantly, and the diagnosis and treatment appeared to be at a dead end. However, atypical cells in the CSF were identified by CSF cytology, and biochemical markers showed significantly elevated levels of protein, LDH, and lactate, suggesting potential malignancy. Experienced morphologists could make a preliminary judgment based on the morphologic characteristics of the atypical cells, combined with the patient’s age of onset and imaging changes, which provided key clues for clinical diagnosis and ultimately clarified the diagnostic direction of medulloblastoma (19). This case shows the importance of CSF cytology in the diagnosis and differential diagnosis of infectious and noninfectious diseases, and also plays a key role in cases with atypical imaging presentations. We conducted a literature review on the use of MRI and CSF cytology for diagnosing LMD. (Table 2) (20–23). Collectively, these studies indicate that while MRI is the primary modality for detecting LMD in medulloblastoma, CSF cytology nevertheless serves as a critical adjunct by identifying occult metastases that are undetectable by imaging. Particularly in patients with atypical MRI presentations or in children, the combined use of both can reduce the rate of missed diagnosis to less than 5% (22). Meanwhile, studies noted that in patients after surgical resection of medulloblastoma, where false-positive MRI occurs due to surgery-related subarachnoid hemorrhage, chemical inflammation, etc., it can be combined with CSF cytology to improve the reliability of the results (11, 24). Although Table 2 effectively outlines the complementary roles and discordance between MRI and CSF cytology in diagnosing LMD through historical studies, several limitations should be acknowledged. First, the included studies span a considerable time period, introducing potential heterogeneity in imaging technologies and cytological interpretation standards. Second, by focusing specifically on discordance rates between these two conventional methods, the table inevitably presents a selective overview that may not fully capture the current diagnostic landscape. Most notably, the diagnostic framework presented does not incorporate emerging molecular techniques such as liquid biopsy, which are redefining the sensitivity of detection.

**Table 2:** Complementary efficacy analysis of MRI and CSF cytology in the diagnosis of medulloblastoma with LMD.

Study	Type of study	Population	Diagnostic performance indicators	Patient outcomes	Core findings	Reference
Fouladi et al., 1999	Cross-sectional study	106 children with medulloblastoma/ primitive neuroectodermal tumor	MRI(+)/CSF(−): 8.5%; MRI(−)/CSF(+): 11.3%	Not reported	CSF cytology and MRI should be routinely used in conjunction to diagnose LMD in patients with medulloblastoma/PNET	(22)
Meyers et al., 2000	Retrospective cohort study	179 patients after resection of medulloblastoma	MRI(+)/CSF(−): 7.3%; MRI(−)/CSF(+): 1.2%	5-year survival for the whole group: 68.0% ± 5.0%; 5-year survival for patients with combined spinal cord tumors: 24.0% ± 8.0%	CSF cytology has clinical value in supplementing MRI missed diagnoses and verifying MRI false positives	(20)
Terterov elt al., 2010	Retrospective cohort study	185 pediatric patients with medulloblastoma	MRI(+)/CSF(−): 4.9%; MRI(−)/CSF(+): 1.1%	Mortality rate in the whole group: 28.7%; lost to follow-up: 24.7%	CSF cytology results combined with neuraxial MRI and may complement deficiencies and reduce the rate of missed dissemination.	(21)
Peeran et al., 2024	Retrospective cohort study	117 medulloblastoma patients	MRI(+)/CSF(−): 4.0%; MRI(−)/CSF(+): 31.6%	Mortality rate in the whole group: 16.2%; mean survival time: 4.6 years	MRI detection of LMD is superior to CSF cytology, but a small number of missed cases remain	(23)

In the diagnostic process of this case, the first CSF examination was performed using a sample of CSF originating from the ventricles of Ommaya’s capsule, in which only a small number of neutrophils were found. Although no heterotypic cells were detected in this specimen, it did assist in ruling out infectious and autoimmune encephalitis. Atypical cells were subsequently found in CSF obtained by a second lumbar puncture, emphasizing the significant impact of the site of CSF collection on cytologic results. Together with the biochemical indices, which also differed, this corroborated that CSF obtained from different puncture sites varied in many ways, and in addition, specimen container selection with potential risk of contamination may further affect the accuracy of the test results (25). Therefore, CSF cytology is highly dependent on the standardization of collection and processing procedures, and improper handling may result in impaired cell morphology, loss of components, or false-negative results (26). For patients with suspected medulloblastoma, CSF samples should be obtained by lumbar puncture as a priority, and standardized collection and processing procedures should be strictly followed to improve diagnostic sensitivity and accuracy.

Beyond conventional imaging and cytological examination, emerging technologies such as proteomics and liquid biopsy are progressively demonstrating their unique value, offering new possibilities for the precise diagnosis of medulloblastoma (9, 27, 28). Proteomics enables the identification of specific protein biomarkers and alterations in signaling pathways associated with tumor pathogenesis through systematic analysis of the protein expression profiles in CSF (29, 30). Simultaneously, liquid biopsy techniques detect nucleic acid biomarkers, including circulating tumor DNA (ctDNA) and RNA in the CSF, working synergistically with proteomics to construct a multi-layered molecular diagnostic map (31, 32). This integrated multi-omics approach provides a more comprehensive characterization of tumor biological features, demonstrating significant advantages in early diagnosis and minimal residual disease detection (33). Furthermore, these molecular profiles offer crucial guidance for prognostic evaluation and identification of potential therapeutic targets (33). Multiple studies have confirmed that in central nervous system tumors, including breast cancer brain metastases and gliomas, CSF analysis of ctDNA and protein markers yields higher detection rates and more complete molecular profiles compared to plasma-based assays (34, 35). Nevertheless, these emerging technologies present certain limitations. Proteomics faces challenges related to technical complexity and data interpretation (36), while liquid biopsy may yield false-negative results in some patients due to insufficient tumor DNA release or specific biological conditions (37, 38). In contrast, traditional CSF cytology maintains its fundamental diagnostic role. Although limited in sensitivity with reported detection rates of 44–67% for LMD in single examinations, improving to 84–91% with repeated sampling (39), it provides irreplaceable diagnostic specificity through direct cytomorphological assessment, proving particularly valuable in resource-limited settings (40, 41). More importantly, cytological examination not only enables risk stratification based on cellular morphological characteristics but also permits evaluation of treatment response through dynamic monitoring of changes in cell quantity and morphology (42, 43). Consequently, an integrated strategy combining the molecular analytical depth of proteomics and liquid biopsy with the morphological specificity of conventional cytology promises to establish a more accurate and comprehensive diagnostic framework. For instance, when cytological results are negative, protein marker or ctDNA analysis can provide molecular evidence supporting the diagnosis of LMD. Conversely, when molecular findings are inconclusive, cytological examination offers crucial morphological confirmation.

Postoperative histopathological examination confirmed the diagnosis of medulloblastoma, anaplastic cell variant, characterized by high cellular density, significant nuclear atypia, and frequent mitotic activity. Immunohistochemical analysis showed positivity for Synaptophysin, weak reactivity for NeuN, and expression of SOX-10, supporting a neuroectodermal origin. The tumor exhibited a high proliferation index, with Ki-67 reaching 40%. Molecular pathology confirmed the presence of MYC gene amplification without MYCN amplification. Together with the anaplastic large-cell morphology, these findings are consistent with a diagnosis of Group 3 medulloblastoma according to the WHO classification, a subtype known for its aggressive behavior and early dissemination (44, 45). Studies have shown that MYC amplification activates signaling pathways such as RAS/MAPK and PI3K/AKT, enhancing tumor cell motility and invasiveness (46). This molecular mechanism predisposes the tumor to diffuse leptomeningeal infiltration rather than the formation of a focal mass, effectively explaining the atypical imaging presentation in this case specifically, the absence of a typical cerebellar vermis mass alongside extensive meningeal enhancement. This case underscores the critical importance of the integrated “imaging-cytology-pathology and molecular” three-tier diagnostic framework in the management of medulloblastoma, particularly in cases with atypical imaging findings. Within this framework, MRI, as the primary imaging modality, although failing to identify a definitive primary lesion, provided essential information on meningeal abnormalities and dissemination patterns, offering crucial spatial localization clues. Subsequently, CSF cytology detected tumor cells, providing direct cytological evidence of LMD and serving as a key bridge linking imaging suspicions to pathological confirmation. Ultimately, histopathological examination established the diagnosis of the anaplastic large cell variant, while molecular pathology, by confirming MYC gene amplification, not only classified the tumor into the high-risk Group 3 molecular subtype but also provided mechanistic insight into its biological behavior, characterized by diffuse dissemination rather than localized growth. This systematic, stepwise diagnostic process successfully resolved the diagnostic challenge posed by the atypical neuroimaging findings, highlighting the necessity of multimodal integrated diagnosis in modern neuro-oncology practice. Looking forward, advances in artificial intelligence-based image analysis are expected to facilitate the development of non-invasive predictive models for molecular subtypes based on multiparametric imaging features. Concurrently, the application of emerging technologies such as CSF liquid biopsy holds promise for the dynamic monitoring of tumor genetic characteristics. Collectively, these developments are poised to advance medulloblastoma diagnosis and treatment toward earlier detection, enhanced precision, and minimal invasiveness, ultimately providing comprehensive support for the formulation of individualized treatment strategies.

In summary, this study reports a pediatric medulloblastoma case with atypical imaging manifestations and, through literature review, underscores the pivotal value of CSF cytology in early diagnosis. We recommend the combined use of CSF cytology for suspected medulloblastoma, particularly in diagnostically uncertain cases with atypical MRI presentations, where lumbar puncture is prioritized for sample collection. Furthermore, this case exemplifies the importance of implementing an integrated “imaging-cytology-pathology and molecular” three-tier diagnostic framework, which necessitates strengthened multidisciplinary collaboration in clinical practice. As a single-case report, the generalizability of our findings requires further validation through larger sample sizes and multi-center studies. Therefore, we plan to pursue additional case collection to further substantiate and refine our conclusions.

## Data Availability

The raw data supporting the conclusions of this article will be made available by the authors, without undue reservation.
